# Does age impact outcomes of retrograde intrarenal surgery in the elderly? Results from 366 patients from the FLEXible ureteroscopy outcomes registry (FLEXOR)

**DOI:** 10.1007/s40520-023-02545-1

**Published:** 2023-09-08

**Authors:** Carlo Giulioni, Carlo Brocca, Vineet Gauhar, Bhaskar Kumar Somani, Ben Hall Chew, Olivier Traxer, Esteban Emiliani, Takaki Innoue, Kemal Sarica, Nariman Gadzhiev, Yiloren Tanidir, Jeremy Yuen‑Chun Teoh, Andrea Benedetto Galosi, Daniele Castellani

**Affiliations:** 1 Urology Unit, Azienda Ospedaliero-Universitaria Delle Marche, Università Politecnica Delle Marche, Via Conca 71, 60126 Ancona, Italy; 2 Department of Urology, Ng Teng Fong General Hospital, Jurong East, Singapore; 3 Department of Urology, University Hospitals Southampton, NHS Trust, Southampton, UK; 4 Department of Urology, University of British Columbia, Vancouver, Canada; 5 Department of Urology AP-HP, Sorbonne University, Tenon Hospital, Paris, France; 6 Department of Urology, Fundación Puigvert, Universidad Autónoma de Barcelona, Barcelona, Spain; 7 Department of Urology and Stone Center, Hara Genitourinary Hospital, Kobe, Hyogo Japan; 8 Department of Urology, Biruni University Medical School, Istanbul, Turkey; 9 Endourology department, Saint-Petersburg State Medical University, Saint-Petersburg, Russia; 10 Department of Urology, Marmara University School of Medicine, Istanbul, Turkey; 11 Department of Surgery, Faculty of Medicine, S.H. Ho Urology Centre, The Chinese University of Hong Kong, Hong Kong, China

**Keywords:** Elderly, Flexible ureteroscopy, Renal stones, Retrograde intrarenal surgery, Laser

## Abstract

**Background:**

There has been a consistent increase in the last decades in prevalence of renal stones in elderly.

**Aims:**

To evaluate outcomes of retrograde intrarenal surgery (RIRS) for renal stones in elderly and factors associated with postoperative complications and residual fragments (RFs).

**Methods:**

Data from 12 centers were retrospectively reviewed. Inclusion criteria: ≥ 75 years, renal stones only, normal renal anatomy. Patients were divided into three groups; Group 1: patients aged 75–79 years; Group 2: age 80–84 years; Group 3: age ≥ 85 years. Multivariable logistic regression analyses were performed to assess factors associated with perioperative complications, sepsis, and RFs.

**Results:**

366 patients were included. There were 189 patients in Group 1, 113 in Group 2, and 64 in Group 3. There was no difference between groups regarding stone features and total surgical time. Median length of stay was significantly longer in Group 3 (6.0 days, vs 2.0 days in Group 2 vs 2.5 days in Group 1, p = 0.043). There was no significant difference in postoperative complications and RFs between the groups. At multivariable logistic regression analysis, female gender (OR 2.82) and maximum stone diameter (OR 1.14) were associated with higher odds of sepsis, while surgical time (OR 1.12) and the use of a reusable ureteroscope (OR 6.51) with overall complications. Stone size (OR 1.23) was associated with higher odds of RFs.

**Conclusion:**

RIRS showed safety and efficacy for kidney stones in elderly patients. Surgical time should be kept as short as possible to avoid higher odds of postoperative complications, particularly in females.

## Introduction

Life expectancy will continue to rise in the next decades, especially in industrialized countries [1]. The probability of life expectancy increasing by at least 65% for women and 85% for men underscores the importance of accurately addressing the needs of the elderly population [2].

Renal stones have seen a steady increase in prevalence over the years, affecting approximately one out of ten individuals worldwide at least once in their lifetime, with a disease recurrence in about 2% of cases, [3]. Numerous studies in the literature have reported a global rise in the incidence and prevalence of urolithiasis in recent decades, particularly in industrialized nations [4]. Clinical observations have revealed that as stone frequency increases, there is also a corresponding rise in prevalence associated with age. Among those over 65 years of age, the reported incidence of stone formation ranges between 9.6% and 16% of all stone patients, with a lifetime prevalence of 14% [5].

According to the European Association of Urology guidelines, retrograde intrarenal surgery (RIRS) is recommended as the first-line treatment for renal stones with a maximum diameter of less than 20 mm [6]. However, there is limited evidence to support the use of RIRS in patients over 80 years of age [7–9]. While age itself is not a disease, it is a significant factor contributing to perioperative complications and adverse outcomes due to the presence of increased comorbidities during surgery [10]. Consequently, many clinicians opt for conservative treatment as the least invasive option, leading to ongoing debates regarding the surgical management of elderly patients.

Currently, there is no universally accepted definition of “elderly” and relying solely on chronological age is generally insufficient to provide a comprehensive definition. Geriatrics recognizes that advancing age is associated with a decline in functional reserve, increased frailty, and greater vulnerability to various health challenges [14]. The majority of individuals aged 75 and older commonly experience multiple pathologies with an average of three or more chronic diseases, and needs for assistance, and approximately 50% of this population is on poly-medications [15]. Therefore, the age of 75 years is considered a valid threshold for defining the elderly population in developed countries [16].

Current data on safety and outcomes of RIRS performed in the elderly are limited to single-center, small series [7–9] which showed good results but with longer operative times and hospital stays as compared to younger age groups [7] and discordant results regarding postoperative complications [8, 9]

This study aims to assess the safety and efficacy of RIRS for treating renal stones in elderly patients while identifying potential predictive factors associated with postoperative complications and stone-free rate (SFR) in a large, multicenter series of patients aged 75 years and older.

## Material and methods

### Included patients

A retrospective analysis was conducted on the FLEXOR database, which was established as part of the TOWER group (Team of Worldwide Endourological Researchers, a research wing of the Endourological Society) initiative [11]. This registry included data from 20 centers comprising a total of 6669 adult patients who underwent RIRS for kidney stones between January 2018 and August 2021. Patients with anomalous kidneys, bilateral procedures, ureteric stones, and planned endoscopic combined intrarenal surgery were excluded. The RIRS procedure was performed according to the standard of care and surgical practices followed by each institute involved in the study. The following data were collected: patient demographics, stone characteristics, intraoperative data, postoperative complications, and stone-free rate (SFR). In the presence of multiple stones, data from the largest stone were gathered. Patients with a positive preoperative urine culture underwent antibiotic treatment according to the susceptibility of the isolated pathogen. Prophylaxis was performed with a single dose of antibiotic chosen based on the local prevalence of pathogens and the antibiotic susceptibility profiles specific to each center. Total surgical time was considered as the time from the start of ureteroscopy to the placement of a ureteral stent. Sepsis was defined as “life-threatening organ dysfunction caused by a dysregulated host response to infection” [12]. Post-procedure evaluations of patients were conducted based on the local standard of care, which involved imaging assessments using KUB X-rays, ultrasound, or non-contrast computed tomography (CT) scan within 3 months. Residual fragments (RFs) were considered RFs ≥ 4 mm since the sensitivity of either plain X-rays and ultrasound for stones smaller than 5 mm is poor [13]. Ancillary treatments post RIRS were performed if RFs were symptomatic if the upper urinary tract was obstructed by fragment(s), or at the discretion of the treating physician. Anonymized pooled data was registered under ethical committee protocol obtained by the Asian Institute of Nephrology and Urology (AINU #08/2022). All patients signed an informed consent to collect their anonymized data.

### Grouping of the study population

For the present study, elderly patients were selected. Patients were divided into three groups based on their age. Group 1 included patients aged 75–79 years; Group 2 patients aged 80–84 years; Group 3 patients aged 85 years and older.

### Statistical analysis

The Kolmogorov–Smirnov test was used to assess continuous variables for normality. Categorical variables are reported as absolute numbers and percentages, while continuous variables as median and interquartile range. Differences between the groups were analyzed using the Chi-square test or Fisher exact test for categorical parameters and the Kruskal–Wallis test for continuous variables.

A multivariable logistic regression analysis was performed to assess factors associated with having RFs. Two further multivariable logistic regression analyses were done to evaluate factors associated with postoperative overall complications and sepsis. Variables that have been previously suggested in the literature to impact RFs [17] and sepsis [18] were included in the models to assess their significance as independent predictors. Predictors were described using odds ratio (OR) and 95% confidence interval (CI). A p-value < 0.05 was considered to be statistically significant. All statistical tests were done using the SPSS software package version 25.0 (IBM Corp., Armonk, NY).

## Results

366 patients were included. There were 189 patients in Group 1, 113 in Group 2, and 64 in Group 3. Table 1 shows patients’ baseline demographics and stone characteristics. There was a statistically significant higher prevalence of females in Group 3 (62.5%, p = 0.021) compared to other groups (43.4% in groups 1 and 2). The majority of patients in each group were first-time stone formers. Upon presentation, there were a significantly higher number of patients presenting with fever in Group 3, whilst significantly more patients presented with elevated creatinine in Group 2. Nevertheless, there was no difference in the number of patients with a positive preoperative urine culture. The number of prestented patients and stone features was similar between the groups.

**Table 1:** Patients’ baseline demographics and stone characteristics

	Group 1 age 75–79 years N = 189	Group 2 age 80–84 years N = 113	Group 3 age ≥ 85 N = 64	p value
Gender, n (%)				**0.021**
Male	107 (56.6)	64 (56.6)	24 (37.5)	
Female	82 (43.4)	49 (43.4)	40 (62.5)	
Ethnicity, n (%)				0.28
Asian	62 (32.8)	39 (34.5)	28 (43.8)	
Non-Asian	127 (67.2)	74 (65.5)	36 (56.3)	
First presentation of stone, n (%)	137 (72.5)	90 (79.6)	55 (85.9)	0.063
Symptoms on presentation^c^, n (%)^&^				
Hematuria	15 (7.9)	8 (7.1)	6 (9.4)	0.314
Pain	79 (41.8)	43 (38.1)	21 832.8)	0.316
Hematuria and pain	16 (8.5)	10 (8.8)	4 (6.3)	0.309
Elevated creatinine^d^	12 (6.3)	22 (19.5)	8 (12.5)	**0.001**
Fever	26 (13.8)	25 (22.1)	21 (32.8)	**0.002**
Incidental finding of stone	23 (12.2)	19 (16.8)	7 (10.9)	0.240
Pre-operative positive urine culture, n (%)	93 (49.2)	54 (47.8)	41 (64.1)	0.07
Pre-stented, n (%)	109 (57.7)	69 (61.1)	46 (71.9)	0.183
Stone characteristics^b^				
HU, median (IQR)	1010 (791–1215)	900 (555–1200)	849 (622–1151.25)	0.127
Multiple stones^&^	84 (44.4)	52 (46)	31 (48.4)	0.982
Size^a^, mm, median (IQR)	8 (6–14)	11.1 (6–15)	8.0 (5–13)	0.467
Location^c&^				
Upper pole	35 (18.5)	21 (18.6)	15 (23.4)	0.773
Mid pole	68 (36.0)	39 (34.5)	28 (43.8)	0.485
Lower pole	83 (43.4)	51(45.1)	34 (53.1)	0.234
Renal pelvis	33 (17.5)	28 (24.8)	11 (17.2)	0.110

Bold value stands for significant p value

*IQR* interquartile range, *HU* hounsfield unit

^a^Largest diameter

^b^The results include stones as per location both for solitary and in those patients where there were reported multiple stones in different locations

^c^More than one choice possible

^d^Defined as creatinine ≥ 1.2 mg/dL

^&^Fisher’s test

There was no difference between the groups regarding anesthesia methods, type, and size of ureteroscope, number of sheathless procedures, type of laser employed, lithotripsy techniques, and laser and total surgical time (Table 2). The need for a basket to extract RFs was significantly more prevalent in Group 3. The postoperative stay was significantly longer in Group 3 (6.0 [2.0–9.0] days, p = 0.043) than in Group 2 (2.0 [2.0–8.25] days) and Group 1 (2.5 [2.0–9.0] days). Overall, the most common stone type was calcium oxalate monohydrate, followed by calcium oxalate dihydrate.

**Table 2:** Intraoperative and postoperative outcomes

	Group 1 age 75–79 years N = 189	Group 2 age 80–84 years N = 113	Group 3 age ≥ 85 N = 64	p value
Type of anesthesia, n (%)				0.129
Spinal anesthesia	9 (4.8)	4 (3.5)	1 (1.6)	
General anesthesia	180 (95.2)	109 (96.5)	63 (98.4)	
Respiratory control, n (%)				0.730
None	87 (48.3)	49 (45.0)	31 (49.2)	
Gated	28 (15.6)	19 (17.4)	12 (19.0)	
Apneic	65 (36.1)	41 (37.6)	20 (31.8)	
Semirigid URS before RIRS, n (%)	73 (38.6)	51 (45.1)	36 (56.3)	**0.036**
Urethral access sheath size, n (%)				0.105
≤ 8 Fr	15 (7.9)	20 (17.7)	8 (12.5)	
> 8 Fr	147 (77.8)	76 (67.3)	44 (68.8)	
No use of sheath	27 (14.3)	17 (15.0)	12 (18.8)	
Ureteroscope type, n (%)				0.265
Reusable	152 (80.4)	95 (84.1)	57 (89.1)	
Disposable	37 (19.6)	18 (15.9)	7 (10.9)	
Size of ureteroscope tip, n (%)				0.076
7 Ch	1 (0.5)	2 (1.8)	0 (0)	
7.5 Ch	69 (36.5)	33 (29.2)	15 (23.4)	
7.6 Ch	1 (0.5)	5 (4.4)	1 (1.6)	
8 Ch	1 (0.5)	0 (0)	2 (3.1)	
8.5 Ch	36 (19.0)	19 (16.8)	15 (23.4)	
9 Ch	52 (27.5)	38 (33.6)	24 (37.5)	
9.5 Ch	8 (4.5)	6 (5.3)	2 (3.1)	
Missing	21 (11.0)	10 (8.8)	5 (7.8)	
Type of laser, n (%)				0.261
Holmium laser	162 (85.7)	104 (92.0)	56 (87.5)	
Thulium fiber laser	27 (14.3)	9 (8)	8 (12.5)	
Power of holmium machine, n (%)				0.286
< 30W	49 (30.2)	42 (40.4)	19 (29.7)	
> 30W	113 (69.8)	62 (59.6)	45 (70.3)	
Lithotripsy technique*, n (%)				0.416
Dusting	67 (35.4)	34 (30.1)	17 (26.6)	
Popcorning	49 (25.9)	22 (19.5)	14 (21.9)	
Fragmentation	34 (18.0)	23 (20.4)	10 (15.6)	
Combination	137 (72.5)	80 (70.8)	51 (79.7)	
Extraction of fragments with a basket^&^, n (%)	64 (33.9)	45 (39.8)	34 (53.1)	**0.030**
Laser time, minutes, mean (standard deviation)	20 (13–34.5)	24 (17–47.5)	35 (14–50)	0.266
Operation time, minutes, median (IQR)	55 (42–80)	60 (39–104)	55 (35–84)	0.278
Intraoperative complications^&^, n (%)				
Pelvicalyceal system bleeding not requiring blood transfusion	3 (1.6)	1 (0.9)	2 (3.1)	0.188
Ureteric injury due to access sheath requiring stenting	2 (1.1)	2 (1.8)	1 (1.6)	0.866
Postoperative stay, days, median (IQR)	2.5 (2.0–9.0)	2.0 (2.0–8.25)	6.0 (2.0–9.0)	**0.043**
Day surgery^&^, n (%)	15 (7.9)	11 (9.7)	3 (4.7)	0.138
Postoperative complications^&^, n (%)				
Fever/Infections requiring antibiotics (Clavien grade 2)	12 (6.3)	4 (3.5)	3 (4.7)	0.550
Hematuria requiring blood transfusions (Clavien grade 2)	4 (2.1)	3 (2.6)	1 (1.6)	0.190
Sepsis requiring ICU admission (Clavien Grade 4)	8 (4.2)	2 (1.8)	1 (1.6)	0.422
Type of stone, n (%)	n = 73	n = 52	n = 34	–
Uric acid	10 (13.7)	7 (13.5)	2 (5.9)	
Calcium oxalate monohydrate	29 (39.7)	22 (42.3)	11 (32.4)	
Calcium oxalate dihydrate	9 (12.3)	9 (17.3)	6 (17.6)	
Struvite	1 (1.4)	5 (9.6)	3 (8.8)	
Mixed	24 (32.9)	9 (17.3)	12 (35.3)	
Post-operative imaging assessment by^a^, n (%)				0.298
CT scan	40 (21.2)	30 (26.5)	16 (25.0)	
X-ray	87 (46.0)	47 (41.6)	36 (56.3)	
Ultrasound	80 (40.0)	36 (31.9)	29 (45.3)	
Combination	72 (38.1)	37 (32.7)	29 (45.3)	
Residual fragments ≥ 4 mm^&^, n (%)	42 (22.2)	30 (26.5)	14 (21.9)	0.650
Ancillary treatment for residual fragments^&^ n (%)	n = 42	n = 30	n = 14	0.679
SWL	2 (4.8)	1 (3.3)	0 (0)	
RIRS	5 (11.9)	9 (30.0)	2 (14.3)	
PCNL	5 (11.9)	0 (0)	0 (0)	
ECIRS	0 (0)	1 (3.3)	1 (7.1)	
Observation alone	30 (71.4)	19 (63.4)	11 (78.6)	

Bold value stands for significant p value

*IQR* interquartile range, different locations

^a^More than one choice possible

^&^Fisher’s test

There was no significant difference in postoperative complications between the three groups (Table 2). The blood transfusion rate was low at 2.1%, 2.6%, and 1.6 in groups 1, 2, and 3, respectively. The rate of infection was also low, with fever/infection requiring antibiotics in 6.3% (group 1), 3.5% (group 2), and 4.7% (group 3). Sepsis occurred in 4.8%, 1.8%, and 1.6% in groups 1, 2, and 3, respectively.

The incidence of RFs was similar among the three groups, ranging from 21.9% in Group 3 and 22.2% in Group 1 to 26.5% in Group 2 (p = 0.65). Roughly 70% of patients with RFs in each group were on observation only within 3 months of RIRS.

At multivariable logistic regression analysis, female gender (OR 2.82 95% CI 1.12–5.98, p = 0.02) and maximum stone diameter (OR 1.14 95% CI 1.03–1.25, p = 0.01) were factors significantly associated with higher odds of sepsis (Table 3), whilst total operation time (OR 1.12 95% CI 1.01–1.19, p = 0.005) and the use of a reusable ureteroscope (OR 6.51 95% CI 2.12–18.01, p = 0.01) were associated with higher odds of overall complications (Table 4). Only maximum stone diameter (OR 1.23 95% CI 1.04–1.31, p = 0.01) was significantly associated with higher odds of having RFs (Table 5).

**Table 3:** Multivariable analysis of predictive factors of sepsis

	OR (95% CI)	p-value
Age group (Reference group 1)		
Group 2	0.83 (0.41–2.88)	0.67
Group 3	0.48 (0.22–2.36)	0.39
Female gender (reference male gender)	2.82 (1.12–5.98)	**0.02**
Positive preoperative urine culture (Reference negative)	0.32 (0.08–1.40)	0.42
Total operation time	1.02 (0.98–1.04)	0.91
Maximum stone diameter	1.14 (1.03–1.25)	**0.01**

Bold character significant p-value

*HU* Thulium fiber laser, *UAS* ureteral access sheath, *OR* odds ratio, CI confidence interval

**Table 4:** Multivariable analysis of predictive factors of overall complications

	OR (95% CI)	p-value
Age group (Reference group 1)		
Group 2	1.17 (0.41–3.03)	0.77
Group 3	1.25 (0.33–3.64)	0.81
Female gender (reference male gender)	1.42 (0.61–3.87)	0.48
Positive preoperative urine culture (Reference negative)	1.21 (0.51–3.49)	0.67
Total operation time	1.12 (1.01–1.19)	**0.005**
Multiple stones (reference single)	1.68 (0.52–4.86)	0.41
Maximum stone diameter	0.96 (0.91–1.11)	0.93
Pre-stented (Reference non pre-stented)	0.84 (0.26–2.41)	0.74
Reusable scope (Reference disposable)	6.51 (2.12–18.01)	**0.01**

Bold character significant p-value

*HU* Thulium fiber laser, *UAS* ureteral access sheath, *OR* odds ratio, *CI* confidence interval

**Table 5:** Multivariable analysis of predictive factors of residual fragments ≥ 4 mm

	OR (95% CI)	p-value
Holmium laser (Reference Thulium fiber laser)	4.84 (0.34–21.08)	0.47
Age group (Reference group 1)		
Group 2	1.34 (0.41–4.21)	0.53
Group 3	0.79 (0.17–2.21)	0.72
Female gender (reference male gender)	1.87 (0.63–6.88)	0.27
Total operation time	1.11 (0.99–1.19)	0.30
Multiple stones (reference single)	3.21 (0.59–5.08)	0.21
Maximum stone diameter	1.23 (1.04–1.31)	**0.01**
Pre-stented (Reference non pre-stented)	0.97(0.21–4.43)	0.88
Stone clearance modality (Reference combination)		
Dusting only	0.46 (0.06–4.23)	0.65
Fragmentation only	0.26 (0.13–1.65)	0.77

Bold character significant p-value

*HU* Thulium fiber laser, *UAS* ureteral access sheath, *OR* odds ratio, *CI* confidence interval

## Discussion

An increased occurrence of kidney stones among elderly individuals has led to a rise in prevalence, likely influenced by the combination of escalating kidney stone rates and improved life expectancy [19]. This is in line with our data which showed that more than two third of our patients were first-time stone formers. The surgical management of this condition in the elderly lacks clear recommendations, and safety concerns arise due to the presence of comorbidities. Some studies have demonstrated the feasibility of RIRS for treating renal stones in elderly patients, showing comparable overall complication rates to younger patients [8].

Current guidelines do not have specific recommendations for elderly patients but do not impose age limits on the procedures. However, according to the EAU guidelines, endoscopic procedures should be offered to patients with symptomatic renal stones [6]. In our analysis, we observed a high prevalence of fever and elevated creatinine levels upon presentation in patients aged 85 or above. Worcester et al. reported that elderly individuals exhibit an elevated incidence of morbidity associated with kidney stones, as well as an increased susceptibility to infection and sepsis [20]. Therefore, the necessity of temperature management for older patients due to impaired central temperature regulation, and stone removal may be required in this setting. Specifically, ureteroscopy should be preferred over extracorporeal shock wave lithotripsy for stones smaller than 20 mm, as it offers a higher SFR with shorter treatment duration compared to extracorporeal shock wave lithotripsy, and it is associated with lower morbidity compared to percutaneous nephrolithotripsy [21].

In terms of perioperative outcomes, we observed that Group 3 had a longer hospital stay compared to the other groups, while the overall rate of complications remained similar. Specifically, a urinary tract infection is generally associated with an extended length of stay in older patients [22] but in our analysis, there was no significant difference among groups in post-RIRS infections and sepsis rates even if the sepsis rate was twice in Group 1 compared with other ones. This could be explained by a higher incidence of infectious stones in Group 1 but we do not have stone analysis in all patients to fully support this hypothesis. The longer hospital stay seen in Group 3 may be partially attributed to a slower recovery from anesthesia probably due to the more prevalence of frailty in elderly patients since it has been demonstrated that frailty increases with age [23]. Yet, a prospective observational study involving 215 patients identified age ≥ 70 years (OR = 2.311 [1.096–4.876], p = 0.028) as an independent risk factor for delayed neurocognitive recovery after surgery [24].

The aging process affects both the innate and adaptive components of the immune response, making older individuals more susceptible to infections compared to their younger counterparts. This can primarily be attributed to a higher prevalence of concurrent medical conditions and age-related changes in immune system functions, including phagocytosis, cellular migration, and antibody production [25]. These factors potentially increase the risk of morbidity and mortality [25]. As mentioned in the previous paragraph, perioperative complications associated with infections were found to be comparable among the three groups in our study. These findings align with a recent systematic review on sepsis following RIRS, where the sepsis rate ranged from 0.5% to 11.1%, and the septic shock rate ranged from 0.3% to 4.6% [18]. Among the studies included, none identified elderly age as a risk factor for urosepsis. Therefore, we can argue that the risk of sepsis post-RIRS is not increased in the elderly as we demonstrated in our study.

Another important finding in our study was female gender and the maximum stone diameter as independent factors associated with increased odds of postoperative sepsis. Regarding the former, several factors may contribute to this observation. Firstly, the female gender predominated among patients group aged 85 years and above, who have a higher likelihood of developing multiple chronic diseases [26]. Secondly, post-menopausal women experience a loss of urogenital mucosal tropism due to reduced estrogen production. Consequently, their susceptibility to bacterial colonization increases, as evidenced by a higher incidence of asymptomatic bacteriuria [27]. Then, the etiology of sepsis in this context may be attributed to the ascent of bacteria into the upper urinary tract. Moreover, female patients also have a higher risk of mortality in urosepsis compared to their male counterparts [28]. Therefore, it becomes even more crucial to implement preventive measures to avoid postoperative infections in older females since perioperative complications, which are closely associated with adverse outcomes in the elderly, contribute significantly to heightened surgical morbidity and mortality [29]. In the overall FLEXOR series as well female gender was also reported to have a higher incidence of septic complications [30]. Analyzing the predictive factors of overall complications, the operative time was associated with higher overall complications in our study. Due to the prolonged surgical time, patients may experience prolonged exposure to anesthesia, increased blood loss, and a greater likelihood of tissue trauma. Our results were in line with Juliebø-Jones’ study that found that surgical time was a significant predictor of complications after ureteroscopy in patients aged ≥ 85 years (OR = 1.05 95% CI 1.01–1.09, p = 0.013) [9]. Procedural time is influenced by multiple factors, including the ureteral access sheath use, the presence of a preoperative stent, and the surgeon's expertise [31]. Therefore, the careful planning of the procedure, providing comprehensive patient counseling, and engaging in shared decision-making with the patient is mandatory, according to the patients’ and stone characteristics. It must also be noted as a caution that prolonged lithotomy in the elderly may also cause anesthesia and neuropraxic complications [32] which could also contribute to longer post-operative stay.

The use of reusable scopes was another factor associated with higher odds of having post-RIRS complications. In a multicenter randomized trial, patients who underwent RIRS with disposable flexible ureteroscopes experienced a similar overall complication rate compared to cases treated with reusable scopes (3.3% vs 8.8%, p = 0.05). However, major complications occurred less frequently in the former group (0% vs 2.2%, p = 0.02) [33]. In an in vitro and in vivo comparison between disposable and reusable flexible ureteroscopes, the disposable ones exhibited superior visual clarity in water, flexibility, and irrigation efficiency [34]. These improvements may determine a better laser utilization efficiency, reduced operative time, enhanced precision, and minimized renal pelvis mucosa, probably allowing for a lower procedural time.

In our study, we found the larger the stone the higher the RFs. Stone size greatly influences the endoscopic management of renal stones, despite the advancements in endoscopy technology [33]. In a meta-analysis of randomized control trials, both mini-PCNL and standard PCNL demonstrated higher SFR, at 86%, compared to RIRS which had an SFR of 79%. However, the overall rate of complications was significantly lower following RIRS (11%), as opposed to standard (32%) and mini-PCNL (16%) [35]. Therefore, RIRS emerges as the safest approach, offering an acceptable SFR. Nevertheless, it is worth noting that the extended operative time required for treating large renal stones may harm the morbidity associated with this procedure in the elderly population, as supported by our multivariate analysis.

Our study has several limitations that need to be addressed. Firstly, the retrospective design of the study may introduce bias in patient enrollment, potentially affecting the accuracy and generalizability of our findings. Secondly, the patient's comorbidities were not considered during data acquisition. As previously mentioned, the presence of comorbidities has a significant negative impact on surgical risk, surpassing the influence of chronological age [22]. Whilst it would have been ideal to have a frailty index for our patients, the age of 75 years can be viewed as a valid threshold for defining the elderly considering that most of them have multiple diseases and need for assistance [16]. Finally, the complication period was limited to 30 days. Consequently, a more extended follow-up period would be necessary to assess the full extent of morbidity and mortality burden following RIRS in elderly patients.

## Conclusions

This large, multicenter study showed that even in the elderly RIRS was an acceptable and efficient modality for treating kidney stones. Yet, modern-day RIRS demonstrated a good safety profile in elderly patients even in those aged 85 years and above who did not show an increased incidence of postoperative infectious complications compared to those aged 75–84 years. However, the hospital stay was expectedly longer in the older patients. Surgical time should be kept as short as possible to minimize post-operative complications, especially in women who are at higher risk of sepsis.

## References

[1] United Nations-Population Division. Future life expectancy projections 2022–2100. 2022. https://ourworldindata.org/grapher/future-life-expectancy-projections. Accessed 19 June 2023

[2] Kontis V, Bennett JE, Mathers CD, Li G, Foreman K, Ezzati M (2017). Future life expectancy in 35 industrialised countries: projections with a Bayesian model ensemble. Lancet.

[3] Prattley S, Voss J, Cheung S, Geraghty R, Jones P, Somani BK (2018). Ureteroscopy and stone treatment in the elderly (≥70 years): prospective outcomes over 5-years with a review of literature. Int Braz J Urol.

[4] Knoll T, Schubert AB, Fahlenkamp D, Leusmann DB, Wendt-Nordahl G, Schubert G (2011). Urolithiasis through the ages: data on more than 200,000 urinary stone analyses. J Urol.

[5] Wagner CA (2021). Etiopathogenic factors of urolithiasis Factores etiopatogénicos de la urolitiasis. Arch espanoles de urol.

[6] Skolarikos A, Gambari G, Neisius A et al (2023) EAU guidelines on urolithiasis. Presented at the EAU annual congress Milan 2023. ISBN 978-94-92671-19-6

[7] Emiliani E, Piccirilli A, Cepeda-Delgado M, Kanashiro AK, Mantilla D, Amaya CA, Sanchez-Martin FM, Millan-Rodriguez F, Territo A, Amón-Sesmero JH, Palou-Redorta J, Angerri-Feu O (2021). Flexible ureteroscopy in extreme elderly patients (80 years of age and older) is feasible and safe. World J Urol.

[8] Berardinelli F, De Francesco P, Marchioni M, Cera N, Proietti S, Hennessey D, Dalpiaz O, Cracco C, Scoffone C, Giusti G, Cindolo L, Schips L (2017). RIRS in the elderly: is it feasible and safe?. Int J Surg (London, England).

[9] Juliebø-Jones P, Moen CA, Haugland JN, Gjengstø P, Æsøy MS, Beisland C, Ulvik Ø (2023). Ureteroscopy for stone disease in extremely elderly patients (≥85 years): outcomes and lessons learned. J Endourol.

[10] Hu H, Lu Y, He D, Cui L, Zhang J, Zhao Z, Qin B, Wang Y, Lin F, Wang S (2016). Comparison of minimally invasive percutaneous nephrolithotomy and flexible ureteroscopy for the treatment of intermediate proximal ureteral and renal stones in the elderly. Urolithiasis.

[11] Gauhar V, Chew BH, Traxer O (2023). Indications, preferences, global practice patterns and outcomes in retrograde intrarenal surgery (RIRS) for renal stones in adults: results from a multicenter database of 6669 patients of the global FLEXible ureteroscopy outcomes registry (FLEXOR). World J Urol.

[12] Singer M, Deutschman CS, Seymour C (2016). The third international consensus definitions for sepsis and septic shock (sepsis-3). JAMA.

[13] Viprakasit DP, Sawyer MD, Herrell SD, Miller NL (2012). Limitations of ultrasonography in the evaluation of urolithiasis: a correlation with computed tomography. J Endourol.

[14] Orimo H, Ito H, Suzuki T (2006). Reviewing the definition of “elderly”. Geriatr Gerontol Int.

[15] Kaufman DW, Kelly JP, Rosenberg L (2002). Recent patterns of medication use in the ambulatory adult population of the United States: the slone survey. JAMA.

[16] World Health Organization (2015) Significant loss of functional ability, and care dependence. In: World Rep. Aging Heal. http://apps.who.int/iris/bitstream/handle/10665/186463/9789240694811_eng.pdf;jsessionid=2342855AC115632C5C15C1B21BAC57F9?sequence=1. Accessed 24 May 2023

[17] Skolarikos A, Gross AJ, Krebs A (2015). Outcomes of flexible ureterorenoscopy for solitary renal stones in the CROES URS global study. J Urol.

[18] Corrales M, Sierra A, Doizi S, Traxer O (2022). Risk of sepsis in retrograde intrarenal surgery: a systematic review of the literature. Eur Urol open Sci.

[19] Duchene DA, Pearle MS, Griebling TL (2014). Stones and endourology in older adults. Geriatric urology.

[20] Worcester E, Parks JH, Josephson MA, Thisted RA, Coe FL (2003). Causes and consequences of kidney loss in patients with nephrolithiasis. Kidney Int.

[21] Matlaga BR, Jansen JP, Meckley LM, Byrne TW, Lingeman JE (2012). Treatment of ureteral and renal stones: a systematic review and meta-analysis of randomized, controlled trials. J Urol.

[22] Lisk R, Uddin M, Parbhoo A, Yeong K, Fluck D, Sharma P, Lean MEJ, Han TS (2019). Predictive model of length of stay in hospital among older patients. Aging Clin Exp Res.

[23] Song X, Mitnitski A, Rockwood K (2010). Prevalence and 10-year outcomes of frailty in older adults in relation to deficit accumulation. J Am Geriatr Soc.

[24] Wang L, Chen B, Liu T, Luo T, Kang W, Liu W (2023). Risk factors for delayed neurocognitive recovery in elderly patients undergoing thoracic surgery. BMC Anesthesiol.

[25] Agarwal S, Busse PJ (2010). Innate and adaptive immunosenescence. Annals of allergy, asthma immunol.

[26] Divo MJ, Martinez CH, Mannino DM (2014). Ageing and the epidemiology of multimorbidity. Eur Respir J.

[27] Mody L, Juthani-Mehta M (2014). Urinary tract infections in older women: a clinical review. JAMA.

[28] Nachtigall I, Tafelski S, Rothbart A, Kaufner L, Schmidt M, Tamarkin A, Kartachov M, Zebedies D, Trefzer T, Wernecke KD, Spies C (2011). Gender-related outcome difference is related to course of sepsis on mixed ICUs: a prospective, observational clinical study. Crit care (London, England).

[29] Turrentine FE, Wang H, Simpson VB, Jones RS (2006). Surgical risk factors, morbidity, and mortality in elderly patients. J Am Coll Surg.

[30] Emiliani E, Sanz-Gómez I, Somani B, Tailly T, Castellani D, Traxer O, Teoh JYC, Chew BH, Keat WOL, Chai CA, Saeed BH, Shrestha A, Soehabali B, Angerri O, Gauhar V (2023). Does gender influence retrograde intrarenal surgery (RIRS) outcomes?.

[31] Lane J, Whitehurst L, Hameed BMZ, Tokas T, Somani BK (2020). Correlation of operative time with outcomes of ureteroscopy and stone treatment: a systematic review of literature. Curr Urol Rep.

[32] Gumus E, Kendirci M, Horasanli K, Tanriverdi O, Gidemez G, Miroglu C (2002). Neurapraxic complications in operations performed in the lithotomy position. World J Urol.

[33] Bozzini G, Filippi B, Alriyalat S, Calori A, Besana U, Mueller A, Pushkar D, Romero-Otero J, Pastore A, Sighinolfi MC, Micali S, Buizza C, Rocco B (2021). Disposable versus reusable ureteroscopes: a prospective multicenter randomized comparison. Res Rep Urol.

[34] Eisel M, Strittmatter F, Ströbl S, Freymüller C, Pongratz T, Sroka R (2020). Comparative investigation of reusable and single-use flexible endoscopes for urological interventions. Sci Rep.

[35] Giulioni C, Castellani D, Somani BK, Chew BH, Tailly T, Keat WOL, Teoh JY, Emiliani E, Chai CA, Galosi AB, Ragoori D, Tanidir Y, Hamri SB, Gadzhiev N, Traxer O, Gauhar V (2023). The efficacy of retrograde intra-renal surgery (RIRS) for lower pole stones: results from 2946 patients. World J Urol.

